# Prenatal stress causes alterations in the morphology of microglia and the inflammatory response of the hippocampus of adult female mice

**DOI:** 10.1186/1742-2094-9-71

**Published:** 2012-04-20

**Authors:** Yolanda Diz-Chaves, Olga Pernía, Paloma Carrero, Luis M Garcia-Segura

**Affiliations:** 1 Instituto Cajal, CSIC, E-28002, Madrid, Spain

**Keywords:** Prenatal stress, Inflammation, Cytokines, Chemokines, LPS, Microglia, TNF-α, IL1β, IL6, IP10, CXCL10, Hippocampus

## Abstract

**Background:**

Stress during fetal life increases the risk of affective and immune disorders later in life. The altered peripheral immune response caused by prenatal stress may impact on brain function by the modification of local inflammation. In this study we have explored whether prenatal stress results in alterations in the immune response in the hippocampus of female mice during adult life.

**Methods:**

Pregnant C57BL/6 mice were subjected three times/day during 45 minutes to restraint stress from gestational Day 12 to delivery. Control non-stressed pregnant mice remained undisturbed. At four months of age, non-stressed and prenatally stressed females were ovariectomized. Fifteen days after surgery, mice received an i.p. injection of vehicle or of 5 mg/kg of lipopolysaccharide (LPS). Mice were sacrificed 20 hours later by decapitation and the brains were removed. Levels of interleukin-1β (IL1β), interleukin-6 (IL-6), tumor necrosis factor α (TNF-α), interferon γ-inducible protein 10 (IP10), and toll-like receptor 4 mRNA were assessed in the hippocampus by quantitative real-time polymerase chain reaction. Iba1 immunoreactivity was assessed by immunocytochemistry. Statistical significance was determined by one-way or two-way analysis of variance.

**Results:**

Prenatal stress, per se, increased IL1β mRNA levels in the hippocampus, increased the total number of Iba1-immunoreactive microglial cells and increased the proportion of microglial cells with large somas and retracted cellular processes. In addition, prenatally stressed and non-stressed animals showed different responses to peripheral inflammation induced by systemic administration of LPS. LPS induced a significant increase in mRNA levels of IL-6, TNF-α and IP10 in the hippocampus of prenatally stressed mice but not of non-stressed animals. In addition, after LPS treatment, prenatally stressed animals showed a higher proportion of Iba1-immunoreactive cells in the hippocampus with morphological characteristics of activated microglia compared to non-stressed animals. In contrast, LPS induced similar increases in expression of IL1β and toll-like receptor 4 in both prenatally stressed and non-stressed animals.

**Conclusion:**

These findings indicate that prenatal stress induces long-lasting modifications in the inflammatory status of the hippocampus of female mice under basal conditions and alters the immune response of the hippocampus to peripheral inflammation.

## Background

Epidemiological studies in man have shown that impaired intrauterine growth is associated with an increased incidence of cardiovascular, metabolic and other diseases in adult life [1-3]. In addition, prenatal stress may also increase the susceptibility to disease later in life [3-6]. Maternal stress or infection increases the inflammatory response in their offspring and this is thought to increase the risk of depression, schizophrenia and autism [7]. In this regard, there is a recent appreciation for a direct impact of the immune system on the stress axis and its potential involvement in stress-induced affective disorder onset and progression [7]. Furthermore, maternal hormones and neuromediators released during maternal stress can reach fetal organs and directly influence the ontogeny of immune cells. This may be the case for glucocorticoids, which play a major role in fetal ontogeny and tissue maturation before birth [8,9].

Increased levels of IL-6 and TNF-α have been detected in pregnant women reporting high stress and these proinflammatory cytokines are implicated in the development of preeclampsia and premature labor and delivery [10-12]. Stress during pregnancy seems to alter the immune function of the mother [11] and the immune responses in mononuclear cells from cord blood [13], but whether this has any consequences for the immune system and future risk of infectious diseases in the offspring is unknown. However, in a nationwide cohort of all Danish children born from 1977 to 2004, children exposed prenatally to stress had a 25 % and a 31 % increased risk of severe infectious disease and less severe infectious disease hospitalization in childhood, respectively, compared to unexposed children. The susceptibility to infectious diseases in the offspring was highest within the first year of life [14]. In addition, maternal stress has been proposed to have implications for the development of atopic diseases [8]. In fact, pregnant women experiencing stress display alterations in maternal circulating cytokine levels during pregnancy and these increased levels are suspected to be a cause for a higher risk of allergy for the infant later in life [8,15].

Prenatal stress in rats causes long-lasting neurobiological and behavioral alterations, including impaired feedback mechanisms of the hypothalamic-pituitary-adrenal (HPA) axis, disruption of circadian rhythms and altered neuroplasticity [4-6]. However, few studies have examined the consequences of maternal stress on the immune system of the offspring. Nevertheless, it is known that the behavioral response to lipopolysaccharide (LPS) is altered in animal models of maternal stress [8]. For instance, prenatally stressed animals have an enhancement of certain aspects of immune function, including elevated concentrations of pro-inflammatory IL-1β both in the spleen and brain frontal cortex [16] and exhibit augmented fever or high levels of corticosterone [17,18] in response to LPS. In addition, prenatally stressed male rats display an increased pro-inflammatory status when they reach full maturity. Thus, compared to control animals, seven-week-old prenatally stressed rats show increased basal expression of IL-5 that is associated with a slight decrease of IL-2 expression in peripheral blood mononuclear cells, suggesting the existence of a silent pro-inflammatory orientation of the young immune system [19].

Microglia participate in the local inflammatory response of the CNS, releasing a variety of inflammatory mediators, including cytokines such as TNF-α, IL1β and IL6, and chemokines such as interferon γ-inducible protein 10 (IP10; CXCL10) [20-22] that will ultimately cause chronic local inflammation and progressive neurodegeneration [23,24]. In addition, microglia are involved in the regulation of hippocamal neurogenesis [25] and microglial activation and pro-inflammatory cytokines secretion have a detrimental influence on hippocampal neurogenesis [26].

The hippocampus expresses the highest level of glucocorticoid receptors (GRs) within the brain [27,28] and is particularly vulnerable to the effects of stressful experiences [10,29]. Hippocampal GRs regulate the HPA axis by binding glucocorticoids and through negative feedback mechanisms that turn off the HPA response to stressors [30]. In this regard, it has been described that prenatal stress induces structural abnormalities in the hippocampal formation from adolescence until aging, including reduction of neurogenesis in the dentate gyrus across lifespan, which are associated with impairment in hippocampal related spatial tasks [31,32]. Furthermore, prenatal stress alters synaptic communication by changing dendritic morphology and neuronal volume in CA1 of developing rat offspring [33].

In this study, we have assessed whether prenatal stress regulates the number and morphology of microglial cells and the expression of TNF-α, IL1β, IL6 and IP10 in the hippocampus, since this brain region is highly sensitive to the inflammatory response and stress [10,26,29,31-33]. The effect of prenatal stress was assessed, under basal conditions and after an immune challenge caused by the systemic administration of LPS, in female mice that were ovariectomized to eliminate the neuroprotective and anti-inflammatory action of ovarian hormones [34-36]. Since toll-like receptor 4 (TLR4) plays a key role in LPS signaling, we also measured its mRNA levels in the hippocampus of prenatally stressed and non-stressed mice.

## Methods

### Animals

Animals were handled in accordance with the guidelines presented in the UFAW Handbook on the Care and Management of Laboratory Animals and following the European Union guidelines (Council Directives 86/609/EEC and 2010/63/UE). Experimental procedures were approved by our institutional animal use and care committee. Special care was taken to minimize suffering and to reduce the number of animals used to the minimum required for statistical accuracy. Animals were maintained in a temperature controlled room, with 12:12 h light/dark schedule and received food and water ad libitum. Animals used in our experiments were derived from three different reproductions performed at separated seasonal periods throughout the year. Adult virgin C57BL/6 female mice (two months of age) from the Complutense University animal colony were group-housed (six per cage) to coordinate their estrous cycle. Females were then individually housed in the presence of a sexually experienced male C57BL/6 mouse. Pregnant females were then randomly assigned to stress (n = 10) or non-stress (n = 10) groups and individually housed in plastic breeding cages. Stress was started from the gestational Day 12 to parturition. Pregnant females were individually placed in plastic transparent cylinders (3.5 cm diameter, 10 cm long) and exposed to bright light for 45 minutes. Animals were daily submitted to three stress sessions starting at 09:00, 12:00 and 16:00 h, whereas non-stress pregnant females were left undisturbed in their home cages as previously described [37]. Male and female offspring were weaned 21 days after birth, and only offspring from litters containing five to nine pups with similar numbers of males and females were used in the experiments. A maximum of two female pups were taken from each litter to remove any litter effects.

At four months of age, 10 non-stressed and 15 prenatally stressed female mice were bilaterally ovariectomized under halothane anesthesia (Fluothane, AstraZeneca Farmaceutica, Madrid, Spain). Fifteen days after surgery, non-stressed and prenatally stressed female mice received an i.p. injection of vehicle (phosphate-buffered saline, PBS) or an i. p. injection of 5 mg/kg of LPS (from *Escherichia coli* 0111:B4, L2630 Sigma–Aldrich St Louis, MO, USA) dissolved in PBS. Therefore, four groups of animals were generated: non-stressed injected with vehicle (NS-VEH = 5), non-stressed injected with LPS (NS-LPS = 5), prenatally stressed injected with vehicle (PS-VEH = 5) and prenatally stressed injected with LPS (PS-LPS = 10). The dosage of LPS used (5 mg/kg, i.p.) was based on a previous study [38]. Mice were sacrificed 24 hours later by decapitation and the brains were removed. The left hemispheres were immersed in 4 % paraformaldehyde (Sigma-Aldrich) in 0.1 M phosphate buffer, pH 7.4 during 72 hours and then rinsed with phosphate buffer and stored at −20°C in a cryoprotective solution. The hippocampi were dissected from the other halves of the brains and stored at −80°C.

### Real time (RT)-PCR analysis

Interleukin 1β (IL1β), interleukin 6 (IL6), tumor necrosis factor-α (TNF-α), interferon-inducible protein-10 (IP-10) and toll-like receptor 4 (TLR4) mRNA levels were assessed in the hippocampus by quantitative real-time polymerase chain reaction. Tissue was homogenized and RNA was extracted using an illustra RNAspin Mini RNA Isolation Kit (GE Healthcare, Buckinghamshire, UK). First strand cDNA was prepared from RNA using a RevertAidTM H Minus First Strand cDNA Synthesis Kit (MBI Fermentas, Bath, UK) following the manufacturer’s instructions. After reverse transcription (RT), the cDNA was diluted 1:4 (for IL6, IP10 and TLR4) or 1:8 (for TNF-α and IL1β) and 5 μl were amplified by real-time PCR in 15 μl using SYBR Green master mix or TaqMan Universal PCR Master Mix (Applied Biosystems, AB, Foster City, CA, USA) in a ABI Prism 7500 Sequence Detector (AB), with conventional AB cycling parameters (40 cycles of 95°C, 15 s; 60°C, 1 minute). Primer sequences were designed using Primer Express (AB) Primer sequences were designed using Primer Express (AB) and were as follows: for IL1β, forward, 5′-CGACAAAATACCTGTGGCCT-3′ and reverse, 5′-TTCTTTGGGTATTGCTTGGG −3′; for IL6, forward, 5′-GAAACCGCTATGAAGTTCCTCTCTG-3′and reverse, 5′-TGTTGGGAGTGGTATCCTCTGTGA-3′; for TNFα, forward, 5′-GAAAAGCAAGCAGCCAACCA-3′ and reverse, 5′-CGGATCATGCTTTCTGTGCTC-3′; for IP10, forward, 5′-CAGTGAGAATGAGGGCCATAGG-3′ and reverse, 5′-CGGATTCAGACATCTCTGTCTAT-3′; and for TLR4, forward, 5′-GGCTCCTGGCTAGGACTCTGA −3′ and reverse, 5′-TCTGATCCATGCATTGGTAGGT-3′Glyceraldehyde-phosphate dehydrogenase (GAPDH) was selected as control housekeeping gene. GADPH TaqMan probes and primers were the Assay-on-Demand gene expression products (AB). After amplification, a denaturing curve was performed to ensure the presence of unique amplification products. All reactions were performed in duplicate. IL6, IL1β IP10, TNF-α, and TLR4, gene expressions were normalized to GAPDH.

### Immunohistochemistry

Sagittal sections of the hippocampus, 50 μm thick, were obtained using a Vibratome (VT 1000 S, Leica Microsystems, Wetzlar, Germany). Immunohistochemistry was carried out in free-floating sections under moderate shaking. Endogenous peroxidase activity was quenched for 10 minutes at room temperature in a solution of 3 % hydrogen peroxide in 30 % methanol. After several washes in 0.1 M phosphate buffer (pH 7.4), containing 0.3 % BSA, 0.3 % TritonX-100 and 0.9 % NaCl (washing buffer), sections were incubated overnight at 4°C with a rabbit polyclonal antibody for Iba1 (Ionized calcium binding adaptor molecule 1) corresponding to the C-terminus (Wako Chemical Industries, Japan; diluted 1:2000). Primary antibody was diluted in washing buffer containing 3 % normal goat serum. After incubation with the primary antibody, sections were rinsed in buffer and incubated for 2 h at room temperature with biotinylated goat anti-rabbit immunoglobulin G (Pierce Antibody; Rockford, IL, USA; diluted 1:300 in washing buffer). After several washes in buffer, sections were incubated for 90 minutes at room temperature with avidin–biotin peroxidase complex (ImmunoPure ABC peroxidase staining kit, Pierce). The reaction product was revealed by incubating the sections with 2 μg/ml 3,3′-diaminobenzidine (Sigma-Aldrich) and 0.01 % hydrogen peroxide in 0.1 M phosphate buffer. Then, sections were dehydrated, mounted on gelatinized slides and examined with a Leitz Laborlux microscope (Leica Microsystems, Wetzlar, Germany).

### Morphometric analysis

Morphometric analysis was performed by an investigator that was unaware of the identity of the experimental groups. The number of Iba1-immunoreactive cells was estimated with the optical disector method in the hilus of the dentate gyrus of the hippocampus, using total section thickness for disector height at 40× [39,40] and a counting frame of 220 × 220 μm. Section thickness was measured using a digital length gauge device (Heidenhain-Metro MT 12/ND221; Traunreut, Germany) attached to the stage of a Leitz microscope. A total of 28 counting frames were assessed per animal. In addition, the percentage of Iba1 immunoreactive cells with different morphologies was also assessed. Cells were classified in five morphological types: Type I, cells with few cellular processes (two or less); Type II, cells showing three to five short branches; Type III, cells with numerous (>5) and longer cell processes and a small cell body; Type IV, cells with large somas and retracted and thicker processes and Type V, cells with amoeboid cell body, numerous short processes and intense Iba1 immunostaining. For each animal, 120 cells were analyzed in the hilus of the dentate gyrus of the hippocampus.

### Statistical analysis

Data are presented as mean ± SEM. Statistical analyses were performed using GrapdPad Prism5 software (GraphPad Software, San Diego, CA, USA). Main and interactive effects were analyzed by two-way analysis of variance (ANOVA) for repeated or factorial measures or by using the Student’s *t*-test for one-to-one comparisons when appropriate. When justified by the ANOVA analysis, differences between individual group means were analyzed by the *post hoc* test: Newman-Keuls Multiple Comparison test for one way ANOVA and Bonferroni post-test for two-way ANOVA. Differences were considered statistically significant at *P* ≤0.05.

## Results

### Effects of prenatal stress on Iba1 immunoreactivity in the hippocampus under basal conditions

**Figure 1:**
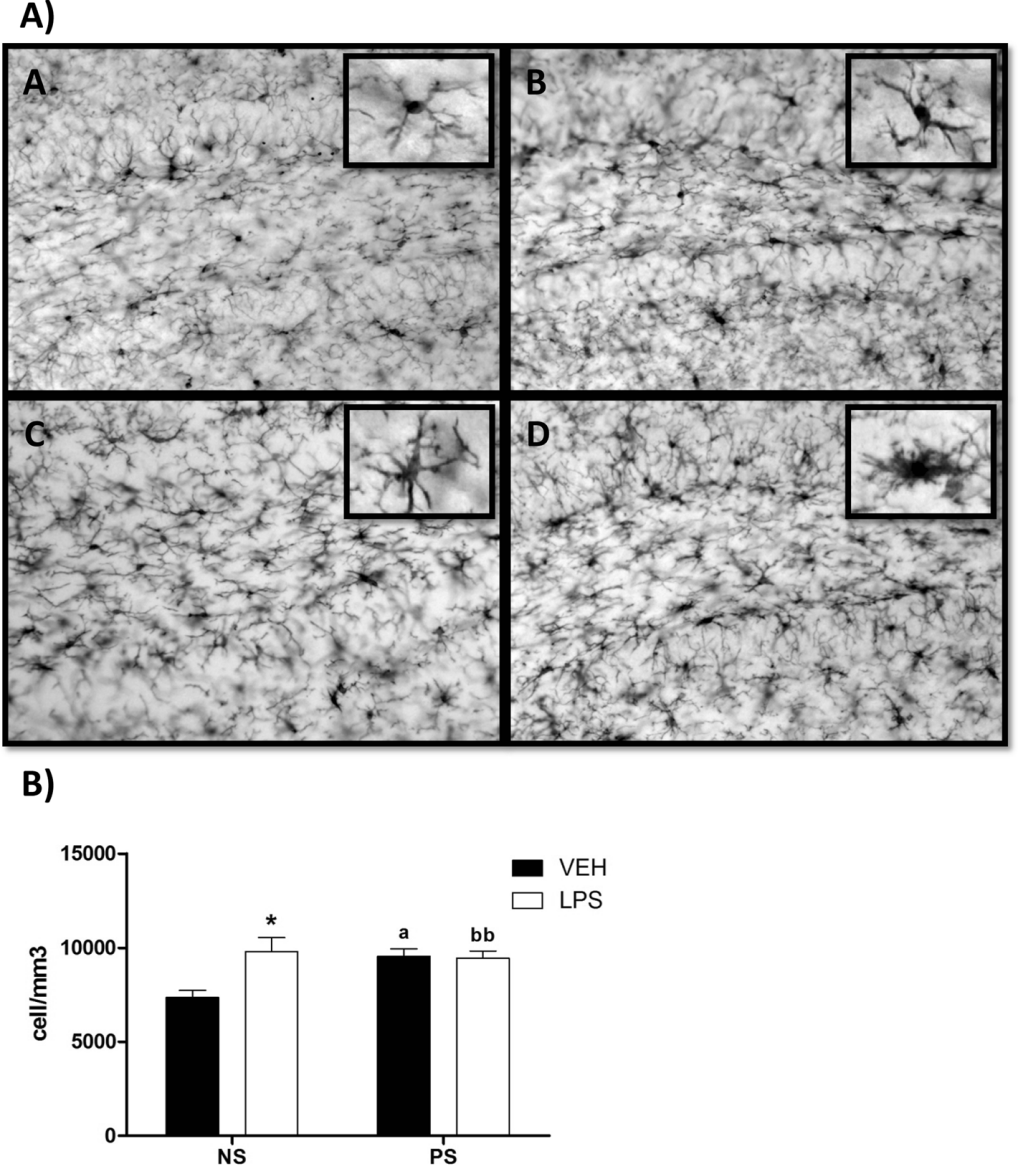
**A) Representative images of the dentate gyrus of hippocampus showing immunoreactivity for Iba1. A)** Non-stressed female treated with vehicle; **B)** Prenatally stressed female treated with vehicle; **C)** Non-stressed female treated with LPS and **D)** Prenatally stressed female treated with LPS. Inserts show details of the morphology of Iba1-immunoreactive cells at high magnification. Scale bar, 50 μm. In the inserts, the scale bar represents 7.5 μm. B) Number of Iba1-immunoreactive cells/mm^3^ in the hilus of dentate gyrus of hippocampus. NS, non-stressed female mice; PS, prenatally stressed females. Filled bars, animals treated with vehicle (VEH). Empty bars, animals treated with LPS. Data are mean ± SEM. *, Significant differences (*P* <0.05) versus NS-VEH. ^a^Significant difference (*P* <0.05) versus NS-VEH and PS-VEH mice. ^bb^Significant difference (*P* <0.01) versus NS-VEH and PS-LPS.

**Figure 2:**
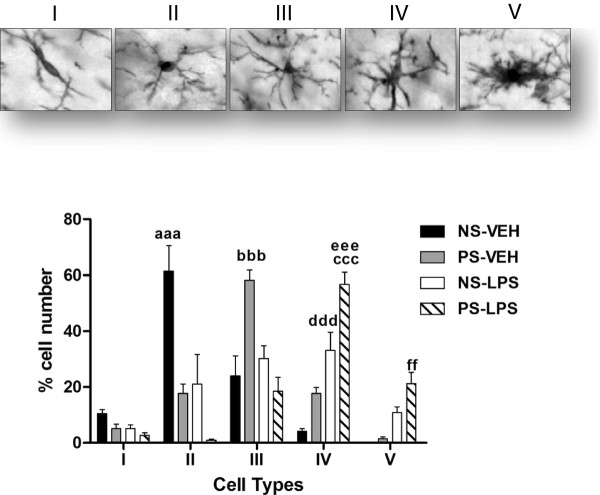
**Morphological changes of Iba1-immunoreactive cells in the hilus of dentate gyrus of hippocampus.** The upper panels show examples of the five morphological types in which Iba1-immunoreactive cells were classified: Type I, cells with few cellular processes (two or less); Type II, cells showing four short branches; Type III, cells with numerous cell processes and a small cell body; Type IV, cells with large somas and retracted and thicker processes and Type V, cells with amoeboid cell body, numerous short processes and intense Iba1 immunostaining. The graph shows the proportion of each morphological type in non-stressed (NS) and prenatally stressed (PS) animals treated with vehicle (VEH) or LPS. aaa, Significant difference (*P* <0.001) of II type cells versus PS-VEH, NS-LPS and PS-LPS mice. bbb, Significant difference (*P* <0.001) of III type cells between NS-VEH, NS-LPS and PS-LPS mice. ccc, Significant difference (*P* <0.001) of type IV cells between NS-VEH mice. ddd, Significant difference (*P* <0.001) of type IV cells versus PS-LPS mice. eee, Significant difference (*P* <0.01) of type IV cells versus PS-VEH mice. ff, Significant difference versus NS-VEH and PS-VEH mice.

Under basal conditions the number of Iba1-immunoreactive cells in the hilus of the dentate gyrus was significantly higher in prenatally stressed animals than in non-stressed animals (one way ANOVA, *P* = 0.02; Newman-Keuls multiple comparison test, *P* <0.05; Figure 1). In non-stressed mice treated with vehicle, the predominant morphology of Iba1-immunoreactive cells was that of a small cell body with three to five cell processes, corresponding to type II cells (61 %; Figure 2). These cells were significantly reduced in prenatally stressed animals treated with vehicle compared to non-stressed mice (two-way ANOVA: There was a significant effect of cell type, F_(4,105)_ = 31.89; *P* <0.0001; no significant group effect, F_(3,105)_ = 0.0003; *P* >0.05 but a significant interaction effect F_(12,105)_ = 23.16; *P* <0.0001. Bonferroni post-test: NS-VEH vs PS-VEH, *P* <0.001). In these animals the predominant morphology of Iba1-immunoreactive cells was type III (58 %). The percentage of type III cells in this group was significantly increased compared to non-stressed mice. (Bonferroni post-test: NS-VEH vs PS-VEH, *P* <0.001)

### Effects of LPS on Iba1-immunoreactivity in the hippocampus

The administration of LPS increased the number of Iba1-immunoreactive cells in non-stressed animals compared to vehicle-injected animals (one way ANOVA, *P* = 0.02; Newman-Keuls multiple comparison test, *P* <0.05; Figure 1). In contrast, LPS administration did not affect the number of Iba1-immunoreactive cells in prenatally stressed animals. However, LPS increased the proportion of Iba1-immunoreactive cells with large somas and retracted and thicker processes (type IV cells) in non-stressed (33 %) and prenatally stressed (57 %) mice compared to vehicle-injected mice (Bonferroni post-test: NS-VEH vs NS-LPS, *P* <0.001; PS-VEH vs PS-LPS, *P* <0.001; Figure 2). Furthermore, LPS increased the proportion of cells with amoeboid cell bodies, numerous short processes and intense Iba1 immunostaining (type V cells) in prenatally stressed animals (21 %) compared to vehicle injected groups (PS-VEH, 1 % and NS-VEH, 0 %) and to non-stressed animals injected with LPS (11 %; Bonferroni posttest: NS-VEH vs PS-LPS, *P* <0.01; PS-VEH vs PS-LPS, *P* <0.01).

### Effects of prenatal stress on IL1β, IL6, TNF-α, IP10 and TLR4 mRNA levels in the hippocampus under basal conditions

**Figure 3:**
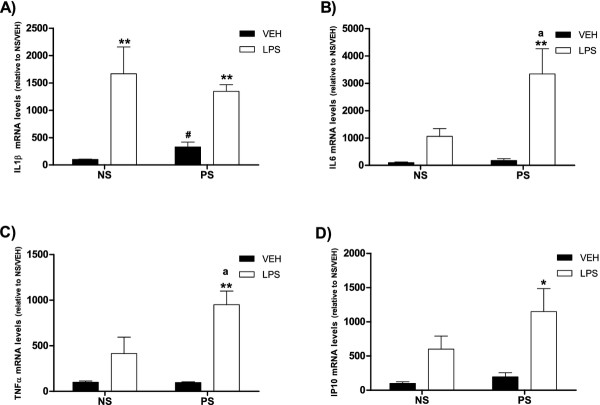
**mRNA levels of inflammatory markers in the hippocampus. A)** Interleukin-1β (IL1β); **B)** Interleukin-6 (IL6); **C)** Tumor necrosis factor α (TNF-α) and **D)** IFN-inducible protein 10 (IP10)**.** NS, non-stressed female mice; PS, prenatally stressed females. Filled bars, animals treated with vehicle (VEH). Empty bars, animals treated with LPS. Data are mean ± SEM. *,**,** Significant differences (*P <0.05, **P <0.01; ***P <0.001) of LPS groups versus their respective VEH groups. a, significant differences (*P* <0.05) versus NS-LPS values. #, Significant difference (*P* <0.05) versus NS-VEH group.

**Figure 4:**
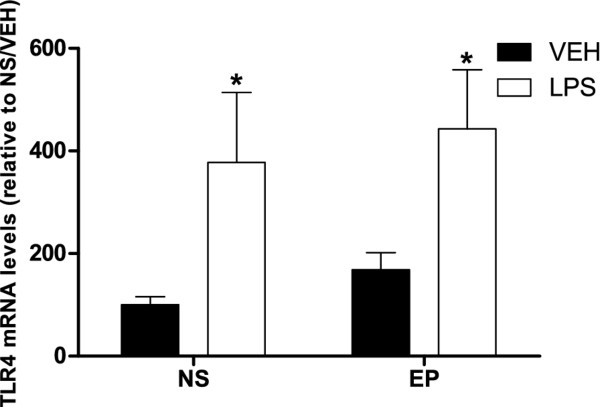
**Toll-like receptor 4 (TLR4) mRNA levels in the hippocampus.** NS, non-stressed female mice; PS, prenatally stressed females. Filled bars, animals treated with vehicle (VEH). Empty bars, animals treated with LPS. Data are mean ± SEM. *, Significant differences (*P* <0.05) of LPS groups versus their respective VEH groups.

Prenatal stress, per se, increased the levels of IL1β mRNA compared to non-stressed mice (*T*-test, *P* = 0.015; Figure 3). In contrast, prenatal stress per se did not affect the mRNA levels for IL6, TNF-α, IP10 (Figure 3) and TLR4 (Figure 4).

### Effects of LPS on IL1β, IL6, TNF-α, IP10 and TLR4 mRNA levels in the hippocampus

The administration of LPS induced a significant increase in the expression of IL1β in both prenatally stressed and non-stressed animals (one way ANOVA, *P* = 0.0002; Newman-Keuls multiple comparison test, *P* <0.01; Figure 3). In non-stressed animals, LPS induced a moderate increase in the mRNA levels for IL6, TNF-α and IP10 that did not reach statistical significance. In contrast, LPS induced a significant increase in the mRNA levels for IL6, TNF-α and IP10 in prenatally stressed mice (IL6, one way ANOVA, *P* = 0.002; Newman-Keuls multiple comparison test, *P* <0.01; TNF-α, one way ANOVA, *P* = 0.0007; Newman-Keuls multiple comparison test, *P* <0.01; IP10, one way ANOVA, *P* = 0.03; Newman-Keuls multiple comparison test, *P* <0.05 Figure 3).

Treatment with LPS significantly increased TLR4 mRNA levels in the hippocampus of both stressed and non-stressed animals compared to mice treated with vehicle (*t*-test, *P* <0.05; Figure 4).

## Discussion

Our results, showing that prenatal stress increases IL1β mRNA levels in the hippocampi of ovariectomized female mice, are in agreement with previous findings for adolescent female rats, which show that prenatal stress increases pro-inflammatory status [19] and elevates splenic and brain IL1β levels [16]. However, a previous study did not detect changes in IL1β levels in hippocampi of prenatally stressed female mice [18]. This discrepancy may be due to the fact that females were ovariectomized in our experiment, therefore, preventing the antiinflammatory effects of ovarian hormones [20,21,34,35].

The increased mRNA levels for IL1β in the hippocampi of prenatally stressed animals detected in the present study may contribute to the increased activity of HPA axis observed in adulthood in these animals [41], since IL1β is known to decrease the affinity of corticosteroid receptors in the hippocampus [42]. In addition, the hyper-secretion of pro-inflammatory cytokines, such as IL1β, is an index of stress and psychopathology [43]. Therefore, the increased expression of IL1β in the hippocampi of prenatally stressed animals may also be related to the depressive-like behaviors observed in these animals [4]. In accordance with these data, it has been described that external stress-induced depression-like behaviors are associated with increased levels of certain cytokines, such as IL1β [44].

In addition, prenatal stress produces an inhibition of neurogenesis in the dentate gyrus of the hippocampi in rats [31,32] and monkeys [45]. In turn, decreased hippocampal neurogenesis related to stress-induced increases in plasma glucocorticoids may be involved in mediating depressive affect [46,47]. Since it has been described that elevation of hippocampal IL1β can markedly suppress hippocampal neurogenesis and since IL1β is induced by stress, it is probable that IL1β mediates the anti-neurogenic effect of stress [26,48-50].

The increase in mRNA levels for IL1β in hippocampi of prenatally stressed animals was accompanied by a significant increase in the number of cells immunoreactive for Iba1, a marker of both resting and reactive microglia. In addition, prenatal stress caused a decrease in the number of Iba1-immunoreactive cells showing three to five short branches (type II) and an increase in the proportion of Iba1-immunoreactive cells with numerous cell processes (type III). This observation is in agreement with accelerated microglial differentiation into a ramified form in prenatally stressed pups of 10 days of age [51]. The significance of these morphological changes in microglia is unknown, but they may reflect a transition of resting microglia towards a pre-activated phenotype. In addition, in prenatally stressed animals there was a higher proportion of Iba1-immunoreactive cells with large somas and retracted and thicker processes (type IV); morphology that is characteristic of activated microglia.

Systemic LPS administration induces peripheral inflammation and central neuroinflammation involving microglial activation, resulting in chronically elevated inflammatory, oxidative and nitrosative stress pathways [38,44]. Induction of the proinflammatory cytokines IL-1β, IL-6 and TNF-α within the CNS leads to a variety of behavioral, physiological and neurological alterations, including fever, diminished feeding behavior, decreased social behavior and decreased exploration (collectively termed “sickness behavior”) [52]. In agreement with this, LPS administration induces fever response in prenatally stressed animals [17,18]. LPS administration also causes deficits in performance and spatial learning and increased corticosterone and IL-1β levels. [18].

Our findings indicate that prenatal stress not only affects mRNA levels for IL1β and Iba1 immunoreactivity in the hippocampus under basal conditions, but also modifies the inflammatory response after the administration of LPS. LPS induced significantly greater increases in the mRNA for IL6, TNF-α and IP10 in hippocampus of prenatally stressed mice compared to non-stressed animals. The enhanced LPS-induced expression of IL6 and TNFα in prenatally stressed animals may increase inflammatory damage [53] and dysfunction of the blood–brain barrier [54]. Furthermore, increased expression of IP10 after LPS could result in differences in the recruitment of T lymphocytes, natural killer cells and monocytes into the central nervous system [55,56]. The expression of these proinflammatory molecules is also associated with increased microglia reactivity [57] and may, therefore, be involved in the higher proportion of Iba1-immunoreactive cells with morphological characteristics of activated microglia (types IV and V) induced by LPS in prenatally stressed animals compared to non-stressed animals. In this regard, although under quiescent condition, microglia may be involved in facilitation of neurogenesis [58]; inflammation-induced microglial activation has been implicated in neurogenesis suppression [59,60]. In addition, the number of activated microglial cells shows a direct correlation with impairment of neurogenesis [61]. Therefore, the increased number of Iba1-immunoreactive cells in the prenatally stressed group could be involved in decreased neurogenesis and in behavioral alterations observed in this animal model [4,31]. Also, the exacerbated increase of IL6 and TNFα observed in prenatally stressed mice after LPS administration could directly affect neurogenesis, since IL-6 and TNFα are known to inhibit neurogenesis [26,62,63].

The inflammatory response to LPS is mediated by TLR4, a member of the IL1 receptor/TLR superfamily that is expressed by astrocytes [64-67] and microglia [68]. In agreement with our results, previous studies have shown that expression of TLR4 is increased in hippocampus of mice after systemic administration of LPS [69,70]. The significance of an increased central expression of TLR4 during systemic inflammation remains to be determined. However, our data show that prenatally stressed and non-stressed mice showed similar changes in the expression of TLR4 in hippocampus after LPS administration. This indicates that the different responses of hippocampus of these animals to LPS are not mediated by differences in TLR4 expression. The different responses of the hippocampi of prenatally stressed and non-stressed animals to LPS may be an indirect effect of deregulation of the HPA axis by prenatal stress [71]. This deregulation of the HPA axis may affect the release of ACTH and glucocorticoids in response to LPS [72-74] and the regulation of the immune response by these molecules [75,76].

## Conclusion

Our findings indicate that the hippocampi of prenatally stressed female mice display a proinflammatory status and display an exaggerated response to an inflammatory challenge in comparison to the hippocampus of female mice that were not submitted to prenatal stress. The different response of the hippocampi of prenatally stressed mice to an inflammatory challenge may predispose these animals to the development of cognitive dysfunction, affective disorders and neurodegenerative diseases.

## Abbreviations

ABC, peroxidase complex, avidin-biotin peroxidase complex; ANOVA, analysis of variance; BSA, bovine serum albumin; CNS, central nervous system; GAPDH, glyceraldehydes-3-phosphate dehydrogenase; GRs, glucocorticoids receptors; HPA axis, hypothalamic-pituitary-adrenal axis; Iba1, ionized calcium binding adaptor molecule 1; IL1β, interleukin-1beta; IL-2, interleukin-2; IL-5, interleukin-5; IL-6, interleukin-6; IP10, interferon γ-inducible protein 10; LPS, lipopolysaccharide; NS-LPS, non-stressed lipopolysaccharide; NS-VEH, non-stressed vehicle; PBS, phosphate buffer saline; PS-LPS, prenatal-stressed lipopolysaccharide; PS-VEH, prenatal-stressed vehicle; RT, reverse transcription; TLR4, toll-like receptor 4; TNF-α, tumor necrosis factor-α.

## Competing interests

The authors declare that they have no competing interests.

## Authors’ contributions

YDC and LMGS designed the experiments and prepared the manuscript. YDC, OP and PC performed the experiments and revised the manuscript. All authors read and approved the final manuscript.
